# Principal component analysis of the Serological response to *Plasmodium Falciparum* using a Multiplex bead-based assay in Nigeria

**DOI:** 10.1038/s41598-024-74236-4

**Published:** 2024-12-28

**Authors:** Jonathan S. Schultz, Mary Okoli, Scott Lee, Colleen M. Leonard, Dean Sayre, Charles M. Heilig, Perpetua Uhomoibhi, Abiodun Ogunniyi, Nnaemeka Ndodo, Nwando Mba, Ado G. Abubakar, Oluwaseun Akinmulero, Ayuba B. Dawurung, McPaul Okoye, Nnaemeka C. Iriemenam, Mateusz Plucinski, Laura Steinhardt, Eric Rogier, Chickwe Ihekweazu

**Affiliations:** 1 Epidemic Intelligence Service, Centers for Disease Control and Prevention, Atlanta, GA USA; 2 Division of Parasitic Diseases and Malaria, Centers for Disease Control and Prevention, Atlanta, GA USA; 3 Centre for Disease Control and Prevention, Abuja, FCT Nigeria; 4 Center for Surveillance, Epidemiology, and Laboratory Services, Centers for Disease Control and Prevention, Atlanta, GA USA; 5 Malaria Branch, U.S. President’s Malaria Initiative, U.S. Centers for Disease Control and Prevention, Atlanta, GA USA; 6 National Malaria Elimination Programme, Abuja, FCT Nigeria; 7 Institute of Human Virology Nigeria, Abuja, FCT Nigeria; 8 Division of Global HIV and Tuberculosis, Centers for Disease Control and Prevention, Abuja, FCT Nigeria

**Keywords:** Plasmodium Falciparum, Nigeria, Serology, Multiplex bead-based assay (MBA), Principal component analysis (PCA), Parasite host response, Epidemiology, Predictive markers

## Abstract

Characterization of serological responses to *Plasmodium falciparum* (*Pf*) is of interest to understand disease burden and transmission dynamics; however, their interpretation is challenging. Dried blood spots from 30,815 participants aged 6 months to 15 years from the 2018 Nigeria HIV/AIDS Indicator and Impact Survey were analyzed by multiplex bead-based assay to measure immunoglobulin G (IgG) to *Pf*-stage-specific MSP-1, AMA-1, GLURPR0, LSA-1, and CSP. These IgG levels were analyzed by principal component analysis (PCA). PC1 and PC2 scores explained 41% and 17% of the total variance, respectively. PC1 unit vectors represented seropositivity. PC2 unit vectors for blood-stage antigens were in opposite directions to liver-stage and sporozoite antigens. PC2 scores were correlated with MSP-1 positively (R = 0.52, P < 0.001) and CSP negatively (R=-0.65, P < 0.001) and may help identify areas with prior exposure but higher risk for increased infections or epidemics. PCA of *Pf* serology can provide summary scores to possibly inform future programmatic interventions.

**Supplementary Information:**

The online version contains supplementary material available at 10.1038/s41598-024-74236-4.

## Introduction

Progress against global malaria has stagnated over the past decade^1^, and as a result the World Health Organization (WHO) now recommends that malaria-endemic countries tailor their interventions on a sub-national scale based on local disease burden and transmission dynamics^2^. This approach aims to maximize intervention effectiveness through more efficient allocation of limited resources in national malaria strategic planning. Effective subnational tailoring depends on accurate measurement of malaria burden. However, accurate burden estimates throughout a malaria-endemic region or country can be challenging to acquire, particularly those at a more granular spatiotemporal scale. Disease burden estimates can provide valuable information to target interventions to intensify control efforts in high burden areas, maintain gains achieved, or implement elimination strategies in low transmission areas^3^.

Various metrics attempt to measure *Plasmodium falciparum* (*Pf*) burden including (1) parasite-based measures such as *Pf* parasite prevalence (*Pf*Pr), (2) clinical infection, severe disease and death incidence from routine data, (3) force of infection (FOI), (4) multiplicity of infection (MOI), (5) seroprevalence and seroconversion rates (SCR) from large cross-sectional surveys, and (6) vector-based measures such as the entomological inoculation rate (EIR) and vectorial capacity of *Anopheles spp*^4–7^. *Pf*Pr from Demographic Health Surveys (DHS) or Malaria Indicator Surveys (MIS) is often considered the gold standard for disease burden, however it needs to be interpreted carefully. These surveys measure cross-sectional *Pf*Pr for active or recent malaria infections using microscopy or rapid diagnostic tests (RDT) and are typically conducted during peak transmission; however, the exact timing of peak cross-sectional *Pf*Pr can vary widely from year to year with changes in vector abundance^8^. Many countries are shifting away from using time- and resource-intensive microscopy during these surveys to RDTs. While easy to perform in the field, the validity of RDTs results is challenged by increasing reports of histidine rich protein-2 (HRP2) deletions, the main molecular target for RDTs, as well as HRP2 persistence after treatment^9^. RDTs also have highly variable limits of detection and RDT positivity can vary greatly between and within surveys and between brands, making results difficult to compare^10^.

Routine data from malaria reporting systems can also be used to characterize malaria burden; however, routine surveillance systems do not represent the true burden of disease in many high burden countries due to asymptomatic infections, self-treatment, and care-seeking outside the public sector^11^. Additionally, incidence of malaria cases reported by routine surveillance data systems can be confounded by many other factors such as changes in access to care, availability of commodities for testing (RDTs or microscopy), population changes, challenges with reporting routine data as well as gathering and integration of complex national data^12^.

Other metrics for malaria burden such as FOI and MOI estimate the number of infections per person per time. FOI counts all patent incident malaria infections (symptomatic or asymptomatic) over time and MOI is the number of concurrent *Pf*clones per host measured by various complex genotyping techniques^5^. Seroprevalence and SCR are determined by collecting data on antibodies directed against a single or limited number of malaria antigens and creating a positivity threshold or fitting simple reverse catalytic models by age groups using maximum likelihood estimates^13,14^. However, there are limitations in detecting small changes in transmission intensity or transmission heterogeneity in a population using these methods^6,13–15^.

Serological profiles to malaria antigens have been a useful tool to measure historical exposure and recent infection due to the differential half-lives and longevity of different antibodies against-malaria stage-specific antigens^15,16^; however, no single antibody or combination of antibodies has been established as a reliable marker of malarial disease burden, transmission dynamics, or populations at risk for epidemics^13,17–22^. In addition, multiplex bead-based assays (MBA) to measure levels of both malarial antigens and antibodies using dried blood spots (DBS) have been developed and validated and are increasingly being used in resource-limited settings to provide detailed serological information^12,23–27^. However, it can be difficult to simultaneously interpret and summarize data on multiple *Pf*-specific antibodies^28^. The objective of this study was to evaluate how multiple antibody levels can be simplified using a principal component analysis (PCA) and results can be used for inferring disease burden and population at risks for epidemics.

For this study, we utilized DBS samples collected from the 2018 Nigeria HIV/AIDS Indicator and Impact Survey (NAIIS) a nationally representative population-based HIV serosurvey^24,26,29–32^. We explored serologic profiles, using a PCA to simplify antibodies levels directed against five different *Pf-*stage-specific proteins: merozoite surface protein-1 (MSP-1), apical membrane antigen-1 (AMA-1), glutamate rich protein-0 (GLURP0), liver stage antigen-1 (LSA-1), and circumsporozoite protein (CSP). Antibody levels and PCA scores were mapped and compared by state. Individual PCA scores were evaluated against known malaria risk factors using univariate and multivariable survey-weighted linear regression models.

## Methods

### Study area

Nigeria is the most populous country in Africa with an estimated population of 223 million in 2023^33^. The country is divided into 36 states and the Federal Capital Territory and organized into six geopolitical zones. All of Nigeria is endemic for *Pf;* the 2018 DHS estimated a national cross-sectional *Pf*infection prevalence of 36.2% by RDT and 22.6% by microscopy in children 6 to 59 months^34,35^. The Sahel of northern Nigeria has seasonal *Pf*transmission while the south has more stable and persistent transmission^36^.

## Nigeria HIV/AIDS indicator and impact survey (NAIIS)

In 2018, the Government of Nigeria conducted the NAIIS through the Federal Ministry of Health (FMoH) and the National Agency for the Control of AIDS, with technical support from the U.S. Centers for Disease Control and Prevention (CDC), Nigeria CDC, and the University of Maryland, Baltimore^37^. The NAIIS was a nationally representative household-based survey to estimate the prevalence and incidence of HIV at state and national levels^29,32,38–40^. Survey methods have been previously described^31^; briefly, a stratified two-stage cluster sample design was used to construct a probability sample of 89,345 households, from which data and venous blood were collected from all consenting individuals aged 15 years and older. From a randomly sampled subset of 28,220 households, blood specimens from all resident children aged 0 to 15 years were also collected following informed consent from their parents. Specimen volume and collection method varied by age: a 14 mL venous blood specimen was collected from adults aged 15–64 years, a 6 mL venous blood specimen was collected from children aged 2–14 years and a 1 mL capillary blood specimen was collected from children aged < 2 years, using a finger stick for children aged 6 to 23 months and a heel stick for infants below 6 months of age. For participants ≥ 2 years who could not provide a venous blood specimen, blood was collected from a finger stick using the 1 ml ethylene diamine tetra acetic acid (EDTA) microtube. Data and blood samples were collected between July and December 2018, with data collection typically completed in less than one month in each state. Whole blood samples were used to create DBS within 24 h of collection and stored at -20 °C; within a week of collection they were transported to a central laboratory and stored at -80 °C^37^.

## Nigeria multi-disease serologic surveillance using stored specimens (nms4)

The NMS4 project was initiated to understand the burden of multiple diseases, including malaria, across all of Nigeria. DBS collected during the NAIIS were analyzed to quantitatively measure levels of *Pf* Histidine Rich Protein-2 (HRP2) antigen and human IgG antibodies directed against *Pf-*specific MSP-1, AMA-1, LSA-1, GLURP0 and CSP using the Luminex MAGPIX™ system (Luminex Corp, Austin, TX) at a target of 50 beads/region; individual median fluorescence intensities (MFI) were reported for each marker^41^. HRP2 results for children from 6 to 59 months of age, non-*Pf*infections, among children under 15 years, and validation of the MBA in Nigeria have been previously described^24,30,31,42^. Children less than 6 months of age were excluded from this analysis to avoid confounding by maternal transplacental IgG. This study analyzed the previously measured raw IgG data.

## Ethical considerations

All participants provided informed consent or assent before enrollment into the NAIIS survey. For children age < 10 years, consent for biomarker testing and consent to future testing was granted by parent or guardian; assent was received from children aged 10–14 years. Laboratory testing for malaria biomarkers as part of the NMS4 project was approved by the National Health Research Ethics Committee of Nigeria (NHREC/01/01/2007) as part of the Government of Nigeria under the Federal Ministry of Health and determined to not involve human research by the Centers for Disease Control and Prevention Human Subjects office (project 0900f3eb819d4c63). All methods were performed in accordance with the relevant guidelines and regulations of the NAIIS survey and NMS4 protocols.

## Multiplex bead-based assay

Purified recombinant antigens were covalently linked to xMAP microspheres (Luminex Corp, Austin, TX), as described previously^43^. The five antigens were utilized for all surveys: *Pf*recombinant MSP-1 (19kD fragment, coupled at pH 5 at 20 µg/mL)^44^; and recombinant AMA-1 (N-terminal region, coupled at pH 5 at 20 µg/mL)^44^, GLURP0 (R0 region peptide coupled at pH 5 at 30 ug/ml)^45^, LSA-1 (Pl1043 epitope peptide, coupled at pH 5 at 60 µg/mL)^41^; CSP (NANP repeat peptide, coupled at pH 5 at 60 ug/mL)^46^. These five *Pf*antigens have been widely used for previous malaria serological studies by multiple groups, and represent the evaluation of long-term IgG responses (MSP1 19kD region, AMA1 N-terminal region)^13,18,47–49^, as well as shorter host IgG responses (GLURP0, CSP and LSA-1)^13,48^. For *Pf* HRP2/3 antigen detection monoclonal IgM and IgG antibodies were covalently bonded to polystyrene BioPlex^®^COOH beads (BioRad; 1715060XX) by the commonly-used EDC/Sulfo-NHS intermediate reaction and previously described in detail^9,10,12,23^.

### Statistical analysis

Descriptive statistics compared the five different *Pf*-specific IgG MFI levels by participant demographics, household characteristics, and state of residence. Continuous MFI levels were dichotomized using Otsu’s method to maximize the inter-class variance by optimizing the separability of the resultant classes as positive or negative for each IgG and HRP2^50,51^. The PCA was used to discover pattens in the data and simplify the multiple IgG levels into composite principal component (PC) scores for each participant accounting for survey design weights (svyprcomp in R). PC unit vectors of the five IgG levels were projected onto PC1 versus PC2 space and PC2 versus PC3 space. Bivariate associations were explored between PC scores and known malaria risk factors such as participant and household characteristics (i.e., age, sex, urban vs. rural location, bed net ownership, number of bed nets, and wealth quintile), and month of sample collection. Correlations between individual PC scores versus individual IgG levels were evaluated. State mean PC1 and PC2 scores were plotted versus state mean HRP2 level and state proportion of urban participants. A survey-weighted linear regression model (svydesign and svyglm in R) was used to compare participant PC scores with traditional malaria risk factors. R version 4.2.2 was used for all analyses.

## Results

### Plasmodium falciparum MBA serological analysis

**Fig. 1:**
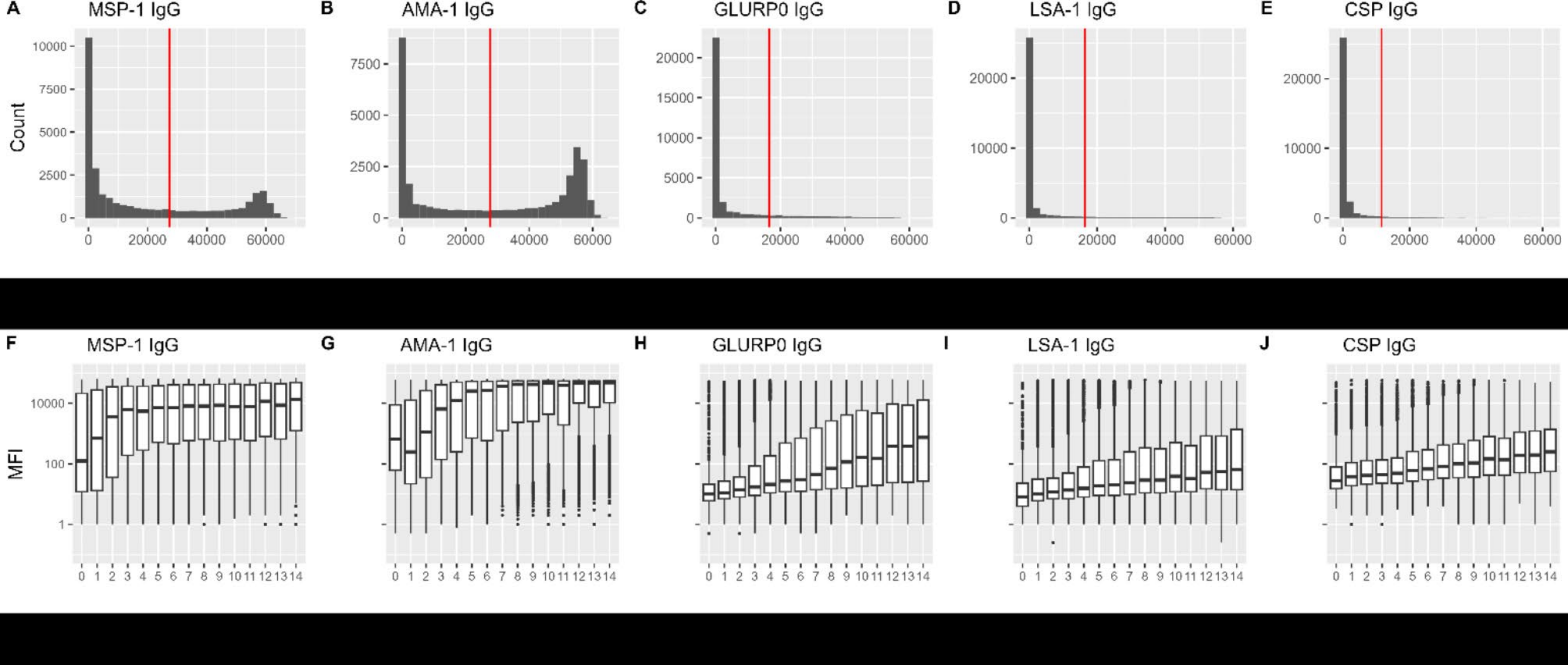
Histograms and age distributions of five IgG antibodies against *Plasmodium falciparum* antigens. (A-E) Histograms with positivity cutoffs (red verticle line) using Otsu’s method and (F-J) box plots of mean fluorescent intensity (MFI) levels of five IgG antibodies against Plasmodium falciparum ( Pf ) antigens ( Pf MSP1, Pf AMA1, Pf LSA1, Pf GLURP0, Pf CSP) by age using a multiplex bead-based assay (MBA) among 30,812 participants aged 6 months to 15 years of age from the Nigeria HIV/AIDS Indicator and Impact Survey (NAIIS) in 2018. Footnote: Boxplot center line represents the median, box edges represent the 25th and 75th percentiles, whiskers extend up to 1.5 * IQR.

**Fig. 2:**
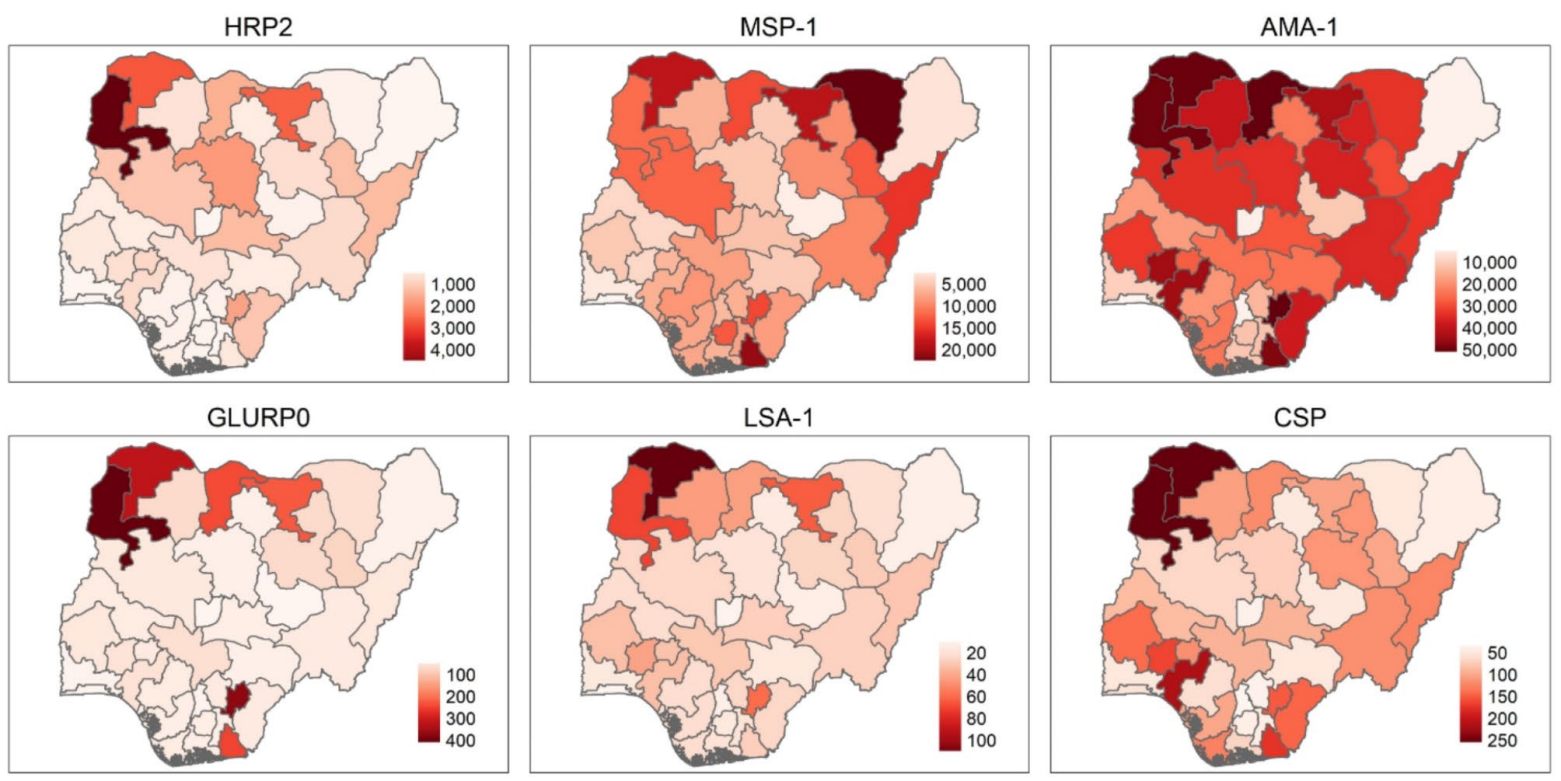
State median histidine rich protein-2 (HRP2) and median IgG levels to *Plasmodium falciparum* antigens (*Pf*MSP1, *Pf*AMA1, *Pf*LSA1, *Pf*GLURP0, *Pf*CSP) from the 2018 Nigeria HIV/AIDS Indicator and Impact Survey (NAIIS).

Among the 206,210 total participants in NAIIS, 31,800 (15.4%) were aged 6 months to 15 years. Of these, data on all malaria-specific IgG levels were available from 30,815; these are included in the analysis. Basic demographics showed that 18,610 individuals (58.5%) lived in rural areas and 11,636 individuals (36.6%) did not sleep under a mosquito net the night prior to the household survey (Table 1). Using Otsu’s method for dichotomization 20.3% (95% CI 19.9–20.8%) were positive for HRP2 antigen, 32.4% (95% CI 31.9% − 32.9%) were positive for anti-MSP-1 IgG, 49.6% (95% CI 49.0% − 50.2%) were positive for anti-AMA IgG, 5.4% (95% CI 5.2% − 5.7%) were positive for anti-GLURP0 IgG, 10.2% (95% CI 9.83% − 10.5%) were positive for anti-LSA IgG, and 2.9% (95% CI 2.7% − 3.0%) were positive for anti-CSP IgG(Supplemental Table 1). Mean MSP-1 and AMA-1 levels were over ten times higher than mean GLURP, LSA, and CSP levels (Fig. 1 and Supplemental Table 1). Levels of all *Pf*-specific antibodies increased with participant age, though at different rates (Fig. 1). The fastest increase was seen for MSP-1 and AMA-1 IgG. Month of DBS sample collection was significantly associated (*p* < 0.001) with all five IgG levels (Supplemental Fig. 1). Median levels of IgG for five antigens showed high geographical variability with the highest mean IgG levels in the Northwest and North Central geopolitical zones (Fig. 2).

**Table 1:** Numbers and characteristics of participants 6 months to 15 years of age among the 2018 Nigeria HIV/AIDS Indicator and Impact Survey (NAIIS).

Characteristic	Total	%
Number of Participants	31,800	100.0
< 5 years	9,231	29.0
5 to < 15 years	22,569	71.0
Female (N, %)	15,579	49.0
Male (N, %)	16,221	51.0
Rural (N, %)	18,610	58.5
Urban (N, %)	13,190	41.5
July (N, %)	2,805	8.8
August (N, %)	5,277	16.6
September (N, %)	6,200	19.5
October (N, %)	6,514	20.5
November (N, %)	6,632	20.9
December (N, %)	4,357	13.7
No bed net used in household	11,636	36.6
One or more bed net used in household	20,156	63.4
*Number of LLIN in household*		
0	11,636	36.6
1	3,732	11.7
2	6,146	19.3
3	3,786	11.9
4	2,853	9.0
5	1,478	4.6
6	919	2.9
7	1,242	3.9
*Wealth Quintile*		
Lowest	6,765	21.3
Second	6,595	20.7
Middle	6,619	20.8
Fourth	6,369	20.0
Highest	5,452	17.1

### Principal component analysis

**Fig. 3:**
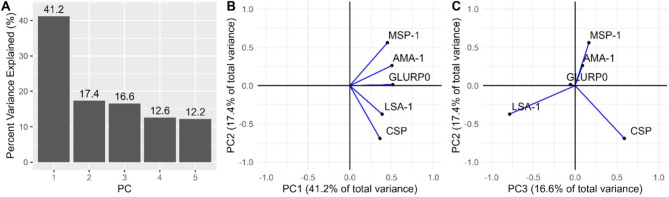
Principal Component Analysis of five anti-*Plasmodium falciparum* IgG levels. (**A**) Percent variance of the mean fluorescent intensity (MFI) levels of five IgG antibodies against Plasmodium falciparum antigens (MSP-1, AMA-1, LSA-1, GLURP0, CSP) explained by each principal component (PC) among 30,812 participants aged 6 months to 14 years of age from the Nigeria HIV/AIDS Indicator and Impact Survey (NAIIS) in 2018. (**B** and **C**) PC loadings for the first three principal components show the PC unit vectors of the mean fluorescent intensity (MFI) levels of five IgG antibodies against Plasmodium falciparum antigens antibodies projected onto PC1 vs. PC2 and PC2 vs. PC3 space.

**Fig. 4:**
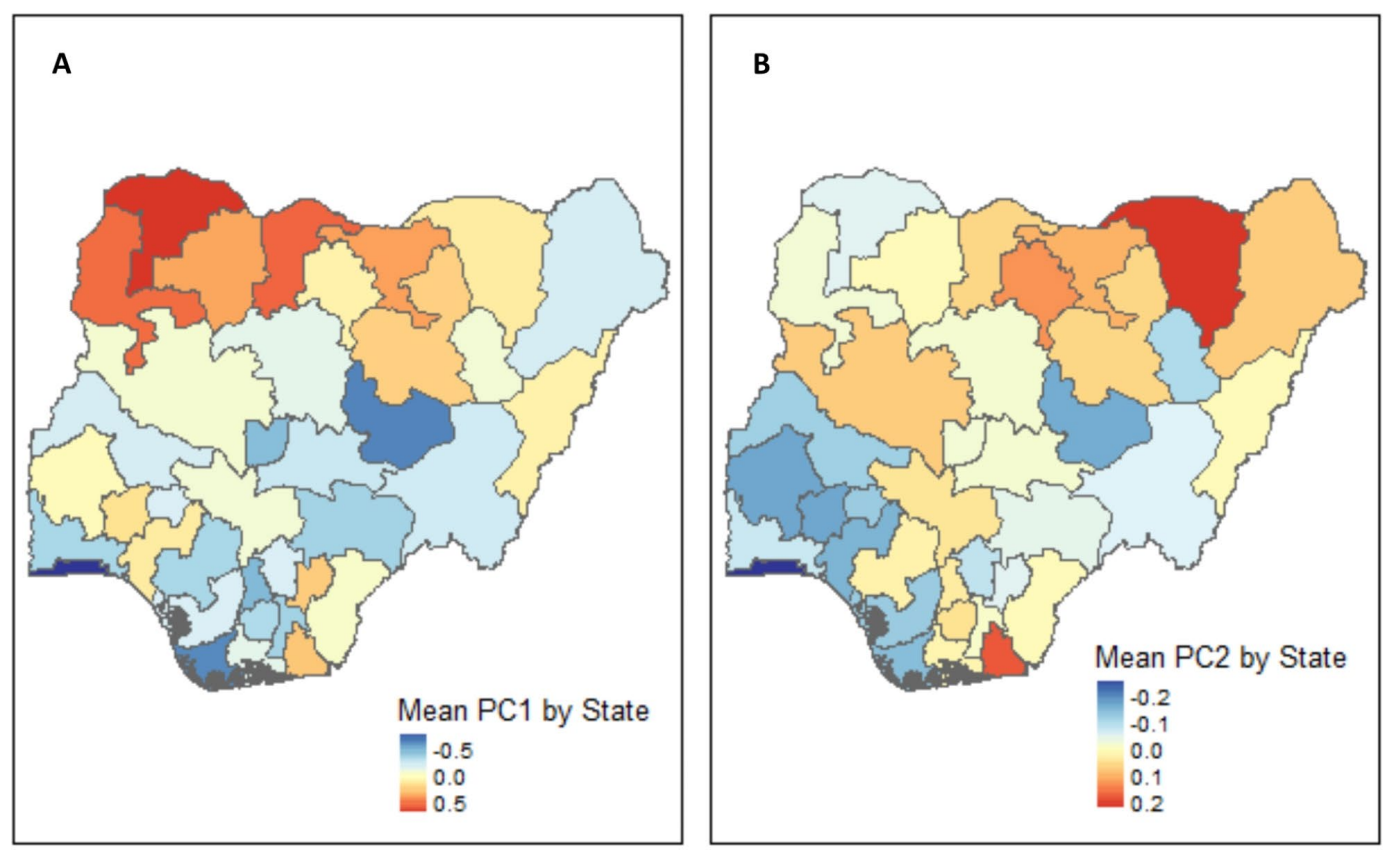
Mean principal component (PC) scores PC1 (**A**) and PC2 (**B**) by state using data from the 2018 Nigeria HIV/AIDS Indicator and Impact Survey (NAIIS) assayed for *Plasmodium falciparum* antibodies.

**Fig. 5:**
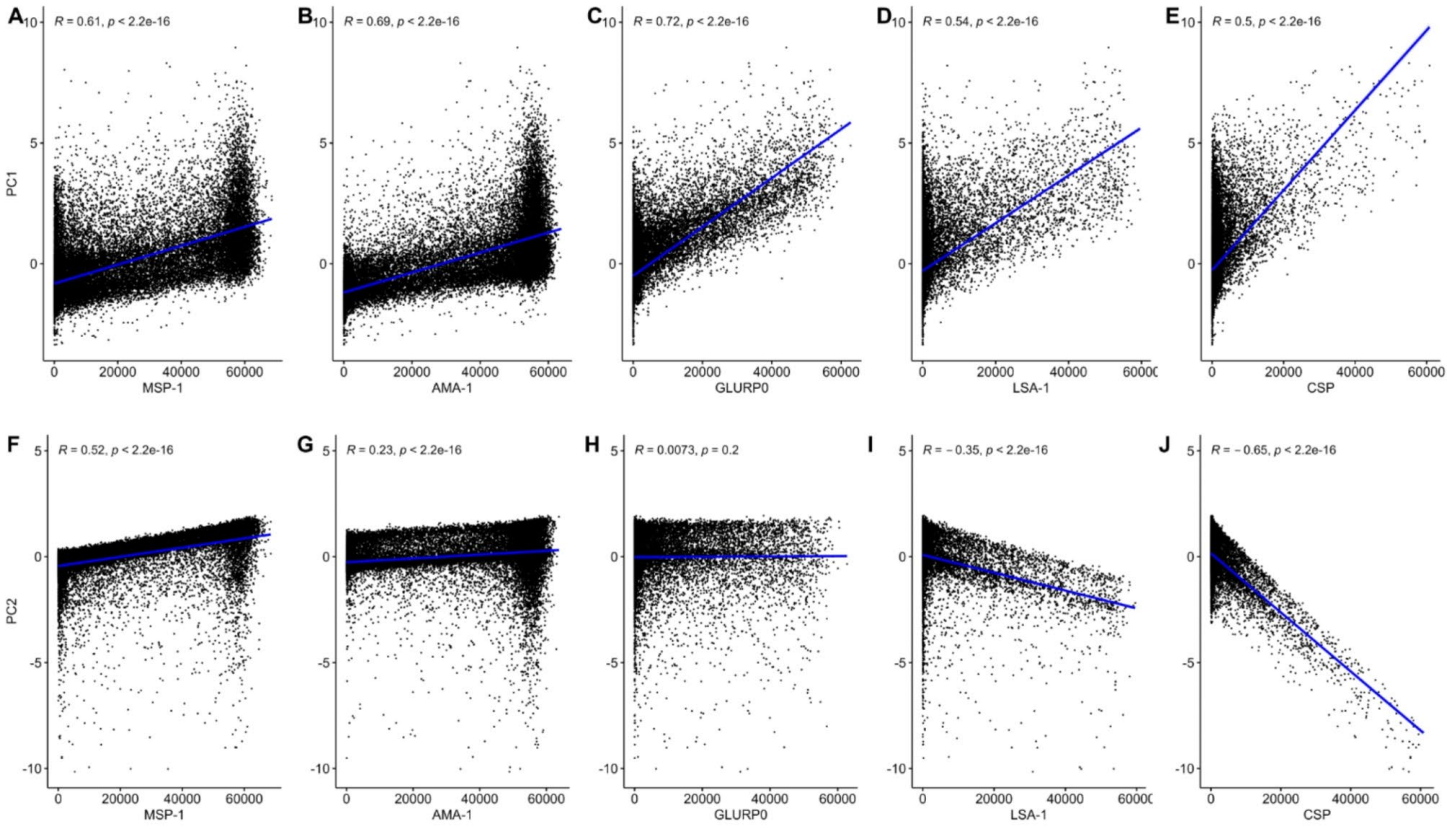
Individual PC1 and PC2 scores versus anti-*Plasmodium falciparum* IgG levels. (**A**–**E**) Individual PC1 and (**F**–**J**) PC2 versus anti-*Plasmodium falciparum* IgG levels (MSP-1, AMA-1, GLURP0, LSA-1 and CSP), with linear regression lines, Pearson correlation coefficients and p-values.

Five PC scores were generated using the five IgG MFI levels for each participant. PC1 accounted for 41.2% of the total IgG variance and PC2 and PC3 explained an additional 17.4% and 16.6% of the variance, respectively (Fig. 3A). PC4 and PC5 accounted for the remaining 12.6% and 12.2% of the total variance in these data, respectively. All PC1 unit vectors on the x-axis had the same directionality and similar magnitudes (Fig. 3B). Individual PC1 and PC2 increased significantly with participant age (Table 2 Supplemental Fig. 2). State mean PC1 score was positively associated with mean HRP2 level (*R* = 0.76, *p* < 0.001) (Supplemental Fig. 3A). State mean PC1 scores were negatively correlated with increasing proportion of urban participants (*R* = − 0.34, *p* = 0.04) (Supplemental Fig. 4A). Individual PC1 scores were positively correlated with all individual *Pf* IgG levels (*R* = 0.61, *R* = 0.69, *R* = 0.72, *R* = 0.54, *R* = 0.50, all *p* < 0.001, for MSP-1, AMA-1, GLURP0, LSA-1 and CSP, respectively) (Fig. 4A, Fig. 5A-E).

**Table 2:** Survey-weighted general linear regression model for univariate and multivariable predictors for individual principal component scores [PC1 and PC2].

**Characteristic**	PC1						PC2					
	**Univariate (PC1)**			**Multivariable (PC1)** *			**Univariate (PC2)**			**Multivariable (PC2)** *		
	**Beta**	**95% CI** ^1^	**p-value**	**Beta**	**95% CI** ^1^	**p-value**	**Beta**	**95% CI** ^1^	**p-value**	**Beta**	**95% CI** ^1^	**p-value**
Age (continuous)	0.11	0.10, 0.11	< 0.001	0.11	0.11, 0.12	< 0.001	0.01	0.00, 0.01	< 0.001	0.01	0.00, 0.01	< 0.001
Age ( > = 5 years)	0.72	0.68, 0.75	< 0.001	—	—	—	0.07	0.05, 0.09	< 0.001	—	—	—
Female	-0.06	-0.10, -0.03	< 0.001	-0.07	-0.10, -0.04	< 0.001	0.00	-0.02, 0.02	0.9	0.00	-0.02, 0.02	> 0.9
Urban Residence	-0.65	-0.72, -0.59	< 0.001	-0.33	-0.41, -0.25	< 0.001	-0.06	-0.10, -0.03	< 0.001	0.00	-0.04, 0.04	0.8
Collection Month *												
July (reference)	—	—	—	—	—	—	—	—	—	—	—	—
August	0.46	0.08, 0.85	0.02	0.39	0.07, 0.72	0.02	-0.14	-0.34, 0.05	0.2	-0.14	-0.34, 0.05	0.15
September	-0.13	-0.31, 0.04	0.13	-0.18	-0.32, -0.04	0.01	-0.16	-0.24, -0.08	< 0.001	-0.05	-0.24, -0.07	< 0.001
October	-0.08	-0.23, 0.06	0.3	-0.11	-0.23, 0.00	0.06	0.03	-0.05, 0.1	0.5	-0.02	-0.05, 0.10	0.5
November	-0.05	-0.17, 0.06	0.4	-0.03	-0.13, -0.07	0.5	0.02	-0.04, 0.08	0.5	-0.02	-0.04, 0.08	0.5
December	0.01	-0.11, 0.12	0.9	0.05	-0.05, 0.14	0.3	-0.04	-0.1, 0.02	0.2	-0.04	-0.10, 0.02	0.2
Net ownership	0.11	0.06, 0.16	< 0.001	—	—	—	0.05	0.02, 0.07	< 0.001	—	—	—
Number of Nets	0.05	0.04, 0.07	< 0.001	0.01	-0.01, 0.02	0.2	0.02	0.01, 0.03	< 0.001	0.01	0.00, 0.01	0.2
Wealth Quintile												
1 (Low)	—	—	—	—	—	—	—	—	—	—	—	
2	-0.32	-0.40, -0.24	< 0.001	-0.27	-0.34, -0.19	< 0.001	0.01	-0.04, 0.06	0.8	0.01	-0.03, 0.06	0.6
3	-0.52	-0.60, -0.43	< 0.001	-0.47	-0.56, -0.37	< 0.001	0.00	-0.05, 0.05	> 0.9	0.00	-0.04, 0.05	0.7
4	-0.84	-0.93, -0.76	< 0.001	-0.72	-0.82, -0.62	< 0.001	-0.04	-0.09, 0.01	0.2	-0.04	-0.06, 0.05	0.7
5 (High)	-1.2	-1.3, -1.2	< 0.001	-1.0	-1.1, -0.91	< 0.001	-0.20	-0.25, -0.15	< 0.001	-0.20	-0.19, -0.07	< 0.001
^1^CI = Confidence Interval. * = Collection month was controlled for by state in univariate analysis. Multivariable model also controlled for state. – = Indicates the reference group for age, collection month, and wealth quintile in the multivariable models.												

PC2 unit vectors on the y-axis grouped antibodies directed against the erythrocytic cycle antigens (MSP-1 and AMA-1) opposite to the sporozoite (CSP) and hepatocyte (LSA-1) antigens. Thus, PC2 represented the approximate difference between an average of MSP-1 and AMA-1 and an average of CSP and LSA-1 (Fig. 3B and C). State mean PC2 scores were not significantly associated with mean state HRP2 level (*R* = 0.16, *p* = 0.34) (Supplemental Fig. 3B), but were negatively correlated with increasing proportion of urban participants (*R* = − 0.33, *p* = 0.04) (Supplemental Fig. 4B). Individual PC2 scores were positively correlated with all individual MSP-1 (*R* = 0.52) and AMA-1 (*R* = 0.23), and negatively associated with LSA-1 (*R* = − 0.35) and CSP (*R* = -0.65) all significant (*p* < 0.001) (Fig. 5F-J). States in the North West and North Central regions had higher mean PC1 scores (Fig. 4A). States with major urban centers of Lagos and Abuja had lower mean PC1 and PC2 scores (Fig. 4A-B and Supplemental Fig. 4A-B).

### Regression analysis of PC scores

In the univariate survey-weighted general linear regression analysis (Table 2), increasing age, male sex, rural residence, net ownership, and number of nets were all significantly associated with higher PC1 scores (*p* < 0.001). Increasing wealth quintile was associated with lower PC1 scores (*p* < 0.001). These relationships were maintained when all were included in the multivariable model. The univariate survey-weighted linear regression model for PC2 showed that increasing age, number of nets, and net ownership were significantly associated with higher PC2 scores (*p* < 0.001). Highest wealth quintile was statistically associated with lower PC2 scores (*p* < 0.001). In contrast, sex was not significantly associated with PC2 scores. When including all predictors in the multivariate model, increasing age, sample collection in September (*p* = 0.01) compared to July (reference), and highest wealth quintile compared to lowest (reference group) remained statistically associated with lower PC2 scores (*p* < 0.001), while adjusting for state.

## Discussion

This study evaluated HRP2 antigen and five *Pf* stage-specific IgG antibodies (MSP-1, AMA-1, GLURP0, LSA-1, and CSP) among 30,815 participants aged 6 months to 15 years of age from one of the largest household surveys ever completed. Utilizing malaria immunology knowledge and PCA, an unsupervised learning technique to identify pattens in these complex and correlated serological data, we were able to simplify five serological levels into two meaningful scores that could be applied to a population level and be informative for public health interventions. These PC summary scores could be helpful to identify areas with the highest *Pf*disease burden (PC1) and evidence of prior but not recent infections (PC2) for targeted interventions recommended in sub-national stratification methods by the WHO^2^. Multiple prior malaria serological surveys in malaria endemic countries could possibly benefit from similar PCA analysis of these complex serological data and provide reproducibility on the general PC conclusions^17–19,41,47^.

### MBA levels and dichotomization

Given the high sensitivity of MBA to detect targets and skewed distribution of serological data, a novel approach using Otsu’s method was used to dichotomize a continuous MFI to determine if a participant was considered positive or negative. Otsu’s method has been primarily used in image thresholding to determine the optimal threshold that provides the largest absolute Student’s t-statistic or maximizing the inter-class variance^52,53^. Creating cutoffs for positivity in serological data can be done using several approaches, including using the mean from known seronegative samples, and adding two or three standard deviations, or using finite mixture models to identify a presumed seronegative sub-population within the sample and then taking the mean plus two or three standard deviations^18^. Finite mixture models were previously used to dichotomize HRP2 levels in children 6–59 months of age resulting in a seropositivity of 38.3% (36.7–39.9%) by NAIIS DBS, however Otsu’s method resulted in 20.3% (19.9-20.8%) which is much closer to the 22.6% (95% CI 21.2–24.1%) via microscopy found in the 2018 DHS^31^, and which may be more clinically relevant in this population. High quality microscopy capacity can be challenging to maintain and can have limited sensitivity missing some infections compared to molecular methods. In addition, advantage of Otsu’s method doesn’t require a set of true negative samples that are often not available^39^ in malaria endemic area. More research is needed to evaluate Otsu’s method or other machine learning techniques to determine the optimal threshold that maximizes the inter-class variance and provides the most relevant cutoffs for malaria serology, however Otsu’s method appeared to be an appropriate in the current study.

### Pc1 interpretation

PCA unit vectors corresponding to the five IgGs projected onto PC1 vs. PC2 space (Fig. 3B) showed that from the PC1 perspective (or x-axis) all the unit vectors were unidirectional suggesting that participants with positive PC1 scores have a higher IgG level or a presence of malaria antibody (prior exposure to malaria). Thus, PC1 described seropositivity as it dichotomized those with and without a serological response to the five measured *Pf* specific IgGs. Individual PC1 scores were positively correlated with all individual *Pf* stage-specific IgG levels and state mean PC1 scores were positively associated with state mean HRP2 MFI levels (*p*< 0.001). As expected, bivariable and multivariable models demonstrated that known malaria risk factors were associated with PC1 or malaria seropositivity. PC1 scores could be interpreted as the percent of the population whose immune system has been exposed to malaria including historic or active and recent infections. PC1 scores may be more important for areas with lower transmission and consideration for elimination strategies as MSP-1 and AMA-1 IgG levels stay elevated in the body for longer periods of time compared to LSA-1 and CSP levels^13,15,16^. Since PC1 represented overall seropositivity, it was associated with other known predictors of prior malaria infections such as increasing age, male sex, living in a rural area, net ownership and number of bed nets owned in univariate and were maintained in multivariable regression models. Geographically the IgG levels and resulting PC1 scores by state showed higher levels in the North West and North Central regional zones which is consistent with median HPR2 levels and previously reported malaria prevalence in 2018^31^.

### Pc2 interpretation

PCA unit vectors from the PC2 (y-axis of Fig. 3B) perspective differentiated antigens expressed primarily during the parasite’s erythrocytic stage (MSP-1 and AMA-1) from those expressed on non-erythrocytic stages, sporozoite (CSP) or by infected hepatocytes (LSA-1). PC2 could thus help differentiate those with prior erythrocytic infection (MSP-1 and AMA-1 response) to those with repeated sporozoite exposure from repeated infectious bites (CSP response) and liver stage infections (LSA-1 response). Areas with higher PC2 scores had higher median levels of MSP-1, but lower levels of LSA-1 and CSP, which require boosting from recent infections, suggesting existing immunity, but less recent infectious bites for boosting. Short half-lives of MSP-1 and AMA-1 of 9.8 days (95% CI 7.6–12.0) have been described in young children with primary infections^54^, however after recurrent boosting in high transmission settings the half-life of MSP-1 has been estimated to be 49.8 years (95% CI 35.4–72.7)^49^. Additionally, seroconversion of MSP-1 and AMA-1 occurs after few infections and is well maintained in the absence of reinfection and may be most useful in areas of low endemicity^16,49,55,56^. Thus, those with higher CSP and LSA-1 responses compared to their MSP-1 and AMA-1 responses are likely being exposed to numerous recurrent infectious bites and resulting infections to mount a robust immune response which represents a marker of more recent infection or recurrent infections^49,57,58^. Importantly, the state mean PC2 scores were not associated with state mean HRP2 MFI levels. Thus, PC2 likely differentiates those with recent or multiple recurrent infections to mount a robust response or higher IgG level to antigens expressed on the sporozoite (CSP) and infected hepatocytes (LSA-1), to those with more remote infections (MSP-1 and AMA-1). Higher PC2 scores thus represent high MSP-1 and AMA-1, but lower LSA-1 and CSP, indicating prior exposures but no recent infections. In addition, dissecting the median difference between MSP-1 and CSP levels by state could possibly be helpful in identifying locations with previous high burden of disease but no recent infections, and therefore at risk of possible epidemics as sorted by the difference of these levels (Supplemental Fig. 5). Higher PC2 scores likely represented prior immunity but lack of recent infections, (high MSP1, low LSA-1, low CSP), and were also associated with known predictors of prior malaria infections such as increasing age, male sex, living in a rural area, net ownership, and number of bed nets owned in univariate analysis. Age, number of bed nets, and highest wealth quintile compared to lowest (reference) remained statistically significant in multivariable regression models of PC2. Mapping of mean PC2 scores by state and graphs of individual PC2 vs. IgG levels also supported the conclusion that those with higher PC2 scores had higher MSP-1, but lower LSA-1 and CSP-1 which require boosting from recent infections. Thus, PC2 scores could be used to identify locations with history of moderate or high endemicity, but possible relatively fewer recent infections. Thus, areas with high PC2 scores could identify epidemic prone areas. Additionally, those areas with high PC1 scores (seropositivity) but low PC2 scores (high LSA-1, high CSP) could benefit from strengthened malaria control strategies. The biological plausibility of PC3 results were challenging to interpret and not included in descriptive or regression analyses (Fig. 3C).

### Limitations

This study was cross sectional but collected samples over seven months. Given the variable and seasonal burden of *Pf* in the Sahel of Northern Nigeria, collecting samples at the same time could have improved the internal validity of this study, but it would have been logistically difficult to implement given the large sample size of this study. To address this concern, geographic location (State) was controlled for in the univariate and multivariable survey-weighted linear regression models. The NAIIS was designed for HIV prevalence estimates and lacked data collection specific to malaria-related behaviors and history of prior infections. The MBA measurements of MSP-1 and AMA-1 likely saturated the assay limits for some participants and could be improved with assay adjustment or serial dilution in future studies. Although the NAIIS provided a large sample size and diverse geographic distribution it is unclear how the PCA would perform in other settings and could result in different PCA interpretations. Lastly, DHS and MIS do not routinely collect DBS samples and thus additional comparisons regarding logistics and costs of sample collection are needed to fully understand the benefit of serological profiles to current approaches.

### Future opportunities

Samples collected during national household surveys, such as the NAIIS, are being increasingly used to evaluate multiple pathogens and allow for collaboration between national surveillance programs. Strengthening laboratory capacity to perform multiplex serological assays could allow additional methods to strengthen surveillance and could help inform national programmatic decisions. Additional research is needed to evaluate using PCA of serological responses to *Pf* in areas of different malaria burden and how PC scores relate to future outbreaks and in low transmission or pre-elimination settings.

## Conclusions

Understanding the burden of malaria in a diverse and heterogenous country like Nigeria is challenging; however, leveraging malaria serology and dimensionality reduction techniques such as PCA can help describe malaria disease burden. Serological responses to multiple *Pf* antigens can clarify the intensity of prior malaria exposures and risk of possible future epidemics in high-risk areas with waning immunity. Further research is needed to evaluate PCA on multiple serological markers of malaria in other countries. However, this exploratory analysis demonstrates the use of PCA on multiple *Pf-*specific antigen responses derived from an MBA that could be used in the future to help direct public health interventions to prevent and control malaria.

## Electronic supplementary material

Below is the link to the electronic supplementary material.

Supplementary Material 1

## Data Availability

Data availability: NMS4 data are owned by the Government of Nigeria (GoN); requests for NMS4 data must be approved by GoN and all other NMS4 principal investigators. Inquiries should be directed to the corresponding authors.
