# Supplementary material for: Principal component analysis of the Serological response to Plasmodium Falciparum using a Multiplex bead-based assay in Nigeria
Source: Sci Rep. 2024 Dec 28;14:30658. doi: 10.1038/s41598-024-74236-4 (PMC11681204; doi:10.1038/s41598-024-74236-4)

**Supplemental Table 1:** Anti-*Plasmodium falciparum* IgG positivity by Otus’s method and distribution statistics of each of the five IgG levels measured by multiplex bead-based assay.

| MBA Target | Total Positive | Percent positive (95% CI) | Mean (SD) | Median (IQR) | Range [Min-Max] |
| --- | --- | --- | --- | --- | --- |
| HRP2 Antigen | 6,262 / 30,824 | 20.3% (19.9% - 20.8%) | 4,541 (7,219) | 376 (68 - 6,184) | [-128 - 29,071] |
| MSP-1 IgG | 9,977 / 30,815 | 32.4% (31.9% - 32.9%) | 19,543 (22,557) | 7,143 (383 - 40,444) | [-1 - 68,516] |
| AMA-1 IgG | 15,280 / 30,815 | 49.6% (49.0% - 50.2%) | 27,448 (24,086) | 26,869 (522 - 53,571) | [-20 - 63,754] |
| GLURP0 IgG | 1,669 / 30,814 | 5.4% (5.2% - 5.7%) | 2,410 (7,904) | 22 (9 - 211) | [-6 - 59,606] |
| LSA-1 IgG | 3,133 / 30,815 | 10.2% (9.8% - 10.5%) | 4,350 (10,298) | 38 (12 - 1,516) | [-10 - 62,754] |
| CSP IgG | 879 / 30,813 | 2.9% (2.7% - 3.0%) | 1,252 (4,376) | 78 (30 - 409) | [-11 - 60,816] |

Totals, percent positive and mean (standard deviation) of MFI, median [interquartile range (IQR)] and range for dried blood spots for Plasmodium falciparum (*Pf*) histidine-rich protein 2 (HRP2) antigen and IgG to *Plasmodium falciparum* (*Pf*) MSP-1, AMA-1, GLURP0, LSA-1, and CSP. Positivity was determined using Otsu’s method for dichotomizing continuous HRP2 antigen and *Pf* IgG antibody levels. 985 / 31,800 participants (3%) of those in our age range did not have all IgG levels.

**Supplemental Figure 1:** Anti-*Plasmodium falciparum* IgG levels by the month of sample collection.


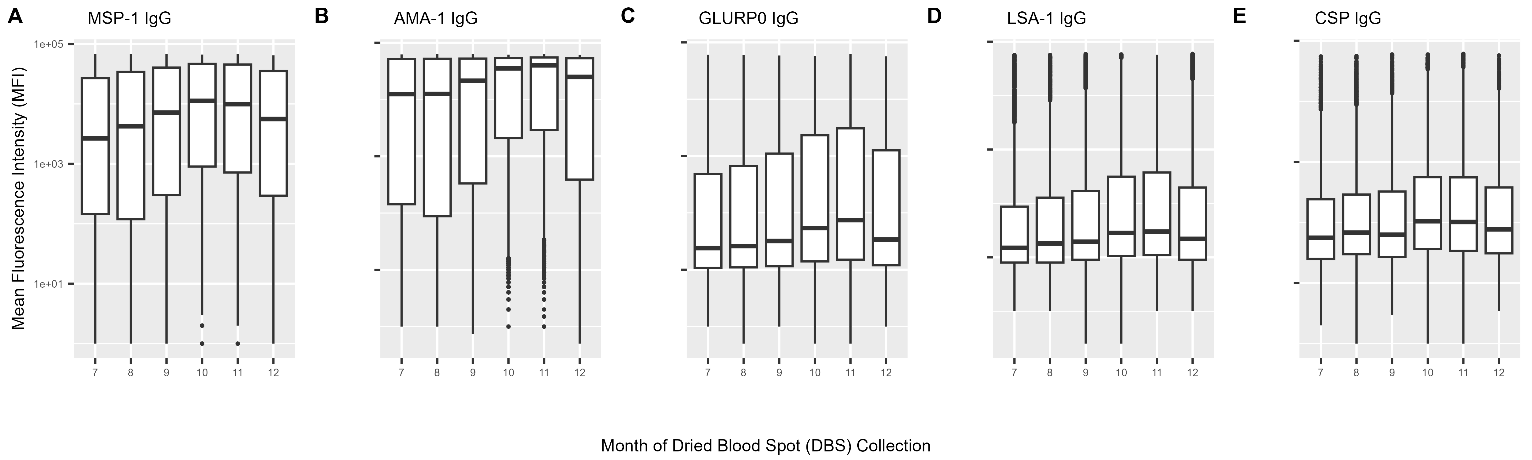


(A – E) Mean fluorescent intensity (MFI) levels of five IgG antibodies against *Plasmodium falciparum* antigens (*Pf*MSP1, *Pf*AMA1, *Pf*LSA1, *Pf*GLURP0, *Pf*CSP) antibodies and *P. falciparum* specific HRP2 by the month of sample collection among 30,812 participants aged 6 months to 14 years of age from the Nigeria HIV/AIDS Indicator and Impact Survey (NAIIS) in 2018. Using the Kruskal-Wallis test, all 5 antibody MFI were significantly different by month of sample collection, p < 0.0001.

**Supplemental Figure 2:** Principal component (PC) scores for PC1 and PC2 by age.


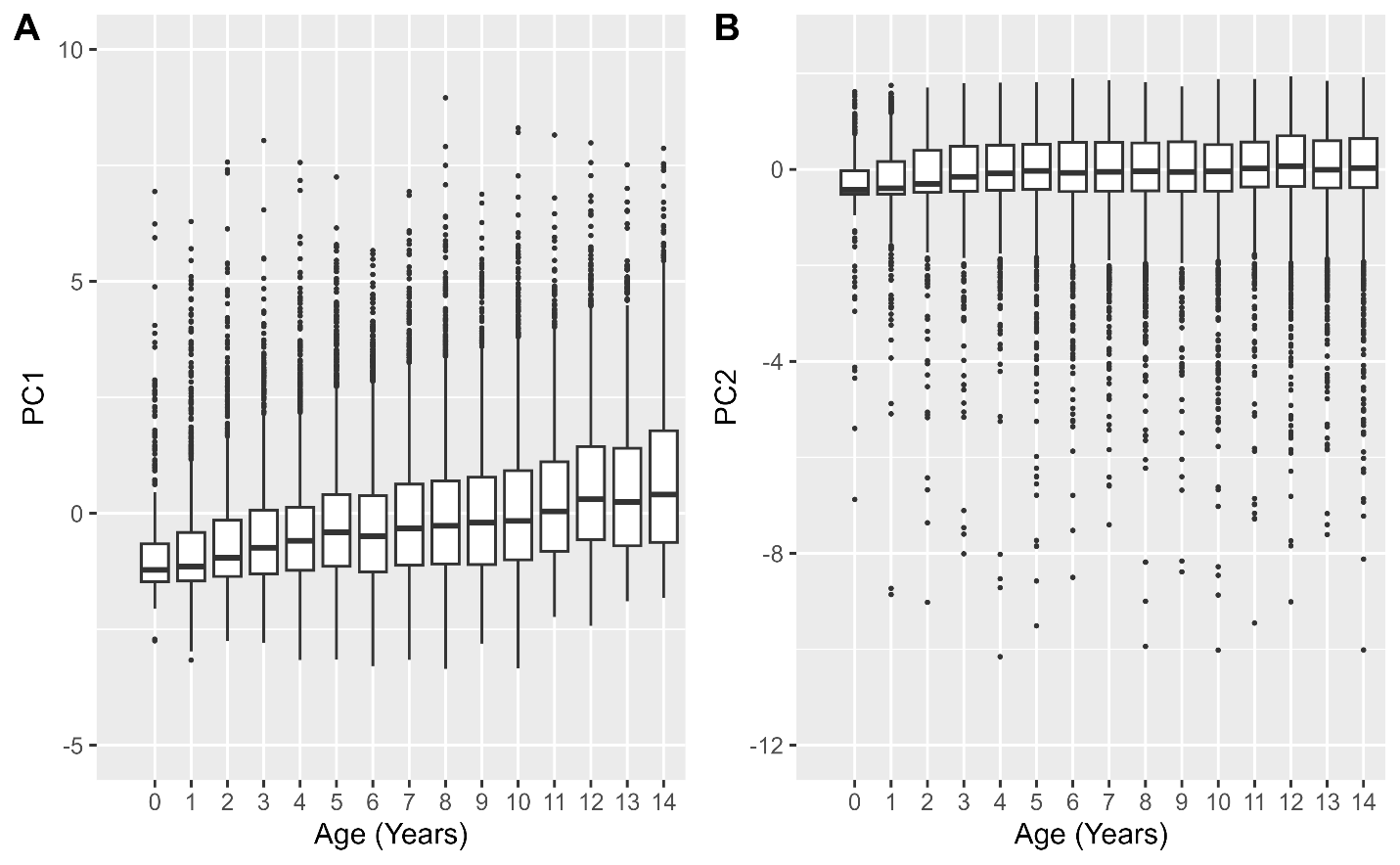


Each boxplot describes the median, interquartile range (25-75% IQR), datapoints past 1.5 times the IQR are represented by individual points among 30,812 participants aged 6 months to 14 years of age from the Nigeria HIV/AIDS Indicator and Impact Survey (NAIIS) in 2018.

**Supplemental Figure 3:** State mean PC1 and PC2 score versus state mean HRP2 level.


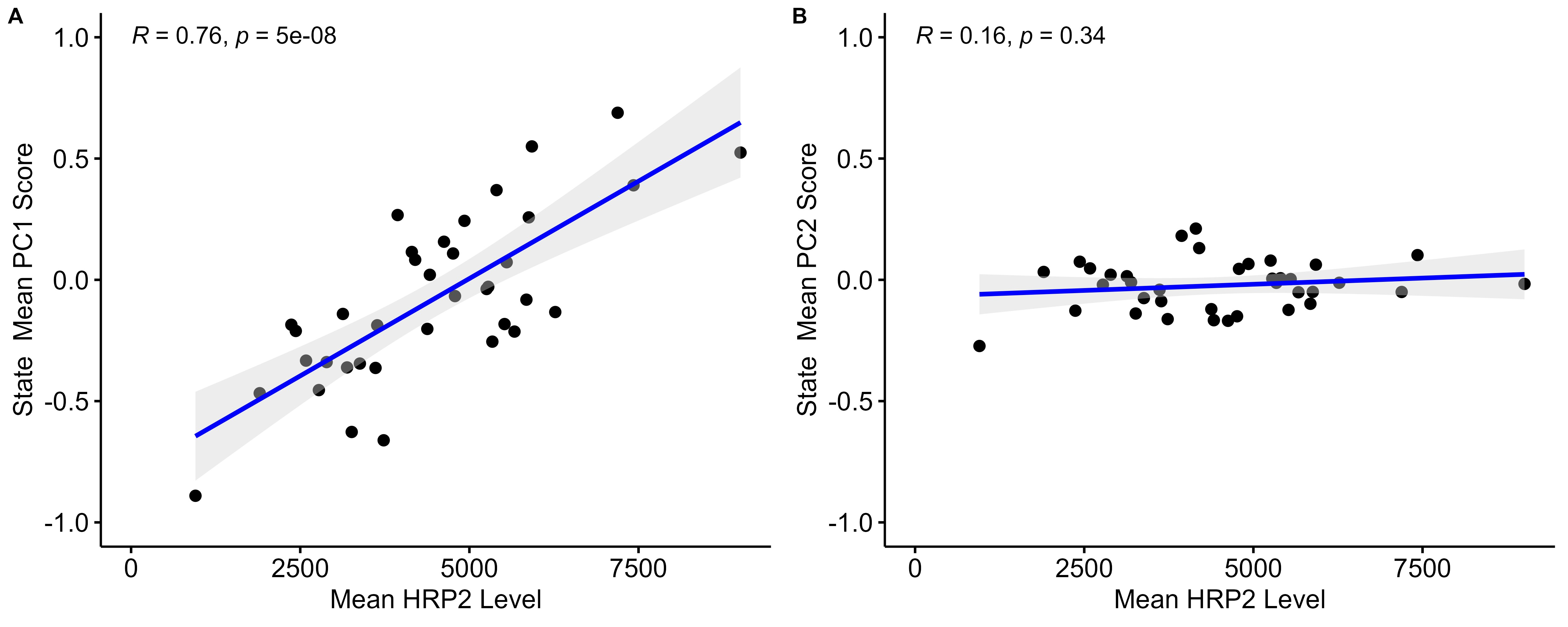


Median fluoresces Intensity level with linear regression line and 95% confidence interval, Pearson correlation coefficient and p-value evaluating correlation of PC scores with burden of recent or active infection.

**Supplemental Figure 4:** Mean State PC1 and PC2 scores versus state urbanicity.


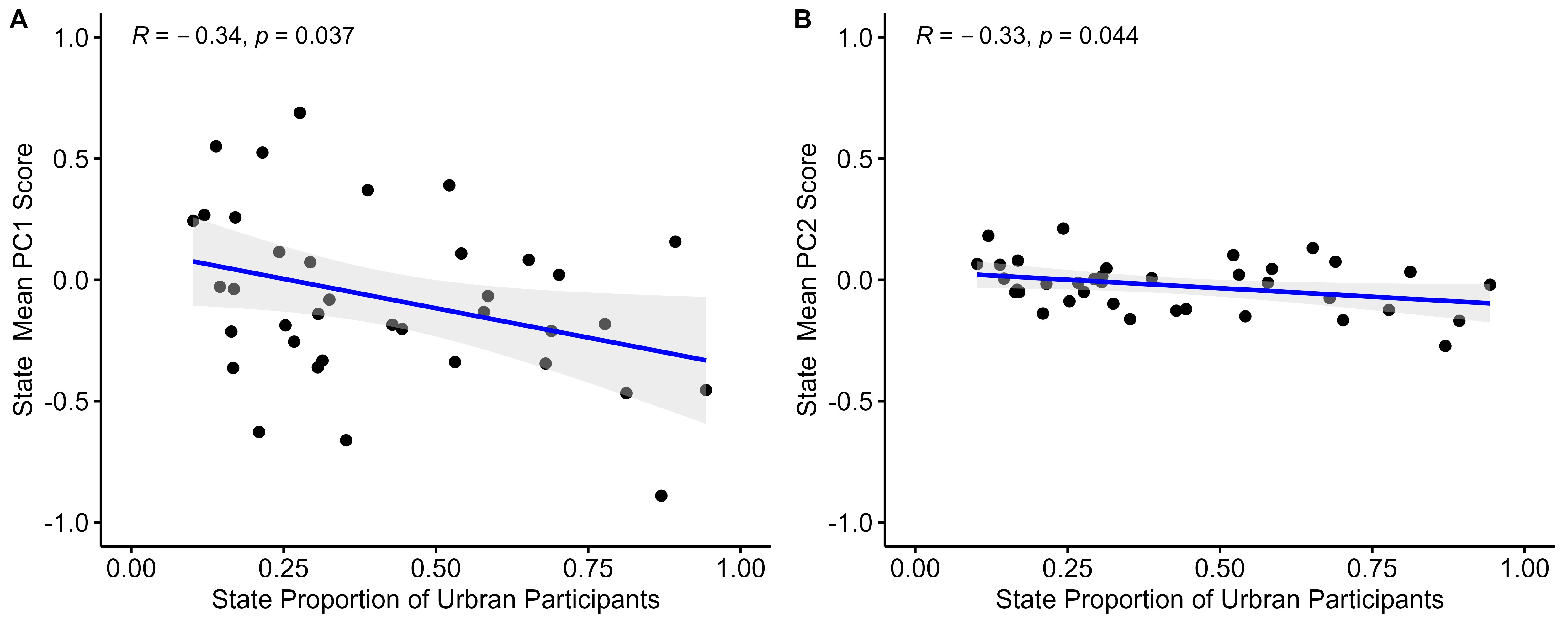


(A) State mean PC1 score and (B) state mean PC2 score versus proportion of urban participants. Each dot represents one state with a linear regression line and 95% confidence intervals.

**Supplemental Figure 5:** State specific boxplots of MSP-1 (red) and CSP (blue) MFI levels sorted from smallest to greatest median differences.


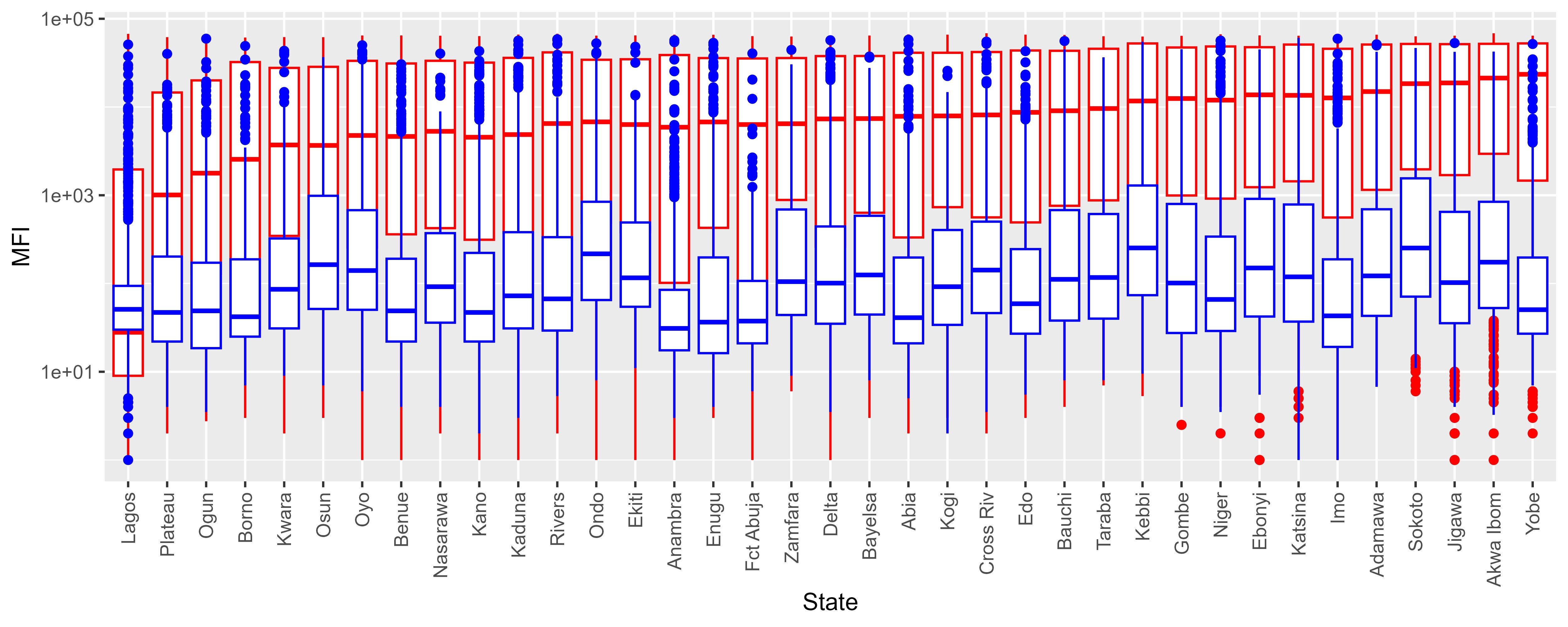

Supplement: Supplementary file 1 — Supplementary Material 1 [file 41598_2024_74236_MOESM1_ESM.docx]
